# Epidemiological findings and policy implications from the nationwide schistosomiasis and intestinal helminthiasis survey in Sudan

**DOI:** 10.1186/s13071-019-3689-z

**Published:** 2019-09-05

**Authors:** Seungman Cha, Mousab Siddig Elhag, Young-Ha Lee, Dae-Seong Cho, Hassan Ahmed Hassan Ahmed Ismail, Sung-Tae Hong

**Affiliations:** 1 Department of Global Development and Entrepreneurship, Graduate School of Global Development and Entrepreneurship, Handong Global University, Pohang, 37554 South Korea; 2 Korea Association of Health Promotion, Gangseo-gu, Seoul, 07653 South Korea; 3 Department of Disease Control, London School of Hygiene & Tropical Medicine, Keppel Street, London, WC1E 7HT UK; 4 Communicable and Non-Communicable Diseases Control Directorate, Federal Ministry of Health, Khartoum, Sudan; 5 Department of Infection Biology and Department of Medical Science, Chungnam National University School of Medicine, Daejeon, 35015 South Korea; 6 Department of Tropical Medicine and Parasitology, Seoul National University College of Medicine, Daehak-ro, Seoul, 03080 South Korea

**Keywords:** Schistosomiasis, Helminthiasis, Mass drug administration, Ecological zone, Sudan, WASH, Cost-effectiveness

## Abstract

**Background:**

The World Health Assembly endorsed the WHO Neglected Tropical Disease (NTD) Roadmap in 2013, in which NTDs were suggested as tracers of equity in the assessment of progress towards the Sustainable Development Goals. Nationwide surveys were undertaken in all 18 states of Sudan to identify the geographical distribution and to estimate the prevalence and intensity of schistosomiasis and other intestinal helminthiases from December 2016 to March 2017.

**Methods:**

We used two-stage random sampling. Each district was subdivided into one to three different ecological zones (EZs) based on proximity to water bodies. Probability-proportional-to-size sampling was used to select schools from each EZ. We estimated schistosomiasis and intestinal helminthiasis prevalence by the centrifugation method and Kato-Katz smears. Multi-level mixed-effect models were used to investigate the relationship between the prevalence of infections and risk factors, including improved water or latrine status at the household or school level. We estimated the cost-effectiveness of a one-time mass drug administration (MDA) intervention with 75% coverage at the district and EZ levels.

**Results:**

A total of 105,167 students from 1772 schools were surveyed. The overall egg-positive rates were: *Schistosoma haematobium*, 5.2%; *S. mansoni*, 0.06%; and intestinal helminths, 5.47%. Severe endemic areas were concentrated in East and South Darfur States. Children living in a house or attending schools with an improved latrine were less likely to be infected with schistosomiasis than those without a latrine (adjusted odds ratio, aOR: 0.45, 95% confidence interval, CI: 0.41–0.51 and aOR: 0.75, 95% CI: 0.70–0.81 at the household or the school levels, respectively). Open defecation was strongly associated with schistosomiasis (aOR: 1.50, 95% CI: 1.35–1.66). In community-wide mass treatment at the district level with an 8% threshold for schistosomiasis, 2.2 million people would not benefit from MDA interventions with 75% coverage despite high endemicity, whilst 1.7 million people would receive the MDA intervention unnecessarily. EZ-level MDA was estimated to be more cost-effective than district-level administration under all circumstances.

**Conclusions:**

Our findings provide updated prevalence figures to guide preventive chemotherapy programmes for schistosomiasis and intestinal helminthiasis in Sudan. Schistosomiasis was found to be common among the inhabitants of fragile and conflict-affected areas. In addition, we found that MDA interventions would be more cost-effective at the sub-district level than at the district level, and there was a strong association between schistosomiasis prevalence and latrine status, at both the household and school levels. This study will help the Sudanese government and its neighbouring countries develop adequate control and elimination strategies.

## Background

Neglected tropical diseases (NTDs) affect 2.7 billion people, most of whom live under the poverty line [1]. The World Health Assembly endorsed the World Health Organization (WHO) NTD Roadmap in 2013, in which NTDs were suggested as tracers of equity in the assessment of progress towards the Sustainable Development Goals [1]. The global health community has witnessed a rekindled interest in NTDs since the 2000s in view of the availability of low-cost and cost-effective interventions [2, 3]. However, NTDs are still most prevalent in disenfranchised communities, where they lead to a vicious cycle of intergenerational transmission of poverty [4–6]. Schistosomiasis and intestinal helminthiasis (IH) affect over 1.5 billion people with limited access to water, sanitation and health services, but the scale-up of preventive chemotherapy remains slow in many parts of the world [7].

Accurate and up-to-date information on schistosomiasis and IH infections is essential for designing and improving national control programmes [7]. Describing the geographical distribution and prevalence of infections also functions as an important tool to promote cooperation and collaboration among various NTD communities nationally and internationally [8–11]. However, the existing data are very outdated in many countries, including Sudan [12].

The cycle of transmission of NTDs cannot be broken by treatment alone, and improvements in water, sanitation and hygiene (WASH) are critical for achieving control and elimination of these diseases [13, 14]. Despite the recognised importance of improved water and sanitation, and their prominence in global strategies, the primary focus of many NTD control programmes in practice has been largely limited to mass drug administration (MDA). Still, evidence on the potential contributions of improvements in WASH on NTDs is scarce [15–17].

In Sudan, it has been estimated that more than eight million people are at risk of infection with schistosomiasis and at least two million people are infected with intestinal worms [18]. The Sudanese government recently prioritised gathering information on the distribution of NTDs, particularly schistosomiasis and soil-transmitted helminthiasis (STH) [19].

The first nationwide mapping [20] for schistosomiasis was conducted in 1986 by the WHO, but it contained a combination of patchwork surveys that were not concurrently carried out. Nationwide data for schistosomiasis and other IHs should be updated to help the Sudanese government make informed policy decisions for the control and elimination of these diseases. Recent surveys [21, 22] in Sudan have tended to be limited to certain geographical areas, and to use *ad hoc* methods, particularly in the selection of states, districts and schools.

For these reasons, nationwide surveys were undertaken in all 18 states to identify the geographical distribution of schistosomiasis and other helminthiases, including IHs, and to estimate their prevalence and intensity, improved water and sanitation coverage at the household and school levels, and the proportion of risk behaviours, such as coming into contact with polluted water and open defecation. This study presents the key findings of these nationwide surveys, along with recommendations for control and elimination programmes, including evidence of associations of disease prevalence with risky behaviours and improved WASH.

## Methods

### Study area

Sudan, the third largest country in Africa, is divided into 18 states and 189 localities (equivalent to districts in other countries), and its population was estimated to be 37.4 million in 2016. There are three main rivers in Sudan: the Nile and its two tributaries, the White Nile and the Blue Nile. Children younger than 15 years account for 45.6% of the total population, while 3.9% are above 59 years of age. As of 2016, the child mortality rate is 83/1000 and life expectancy is 59 years at birth. WASH coverage is low, both for improved drinking water (61%) and improved sanitation (27%) [19]. Students in grades 2, 4 and 6 in 15,761 primary schools comprised the study population, and the entire Sudanese population formed the target population for this study. The study protocol of the nationwide survey was published previously [23].

### Sampling

Two-stage random sampling was undertaken for the nationwide survey. A previous survey [24] suggested that anecdotal data and reports from health care providers or key informants are insufficient to provide adequate information for purposive sampling of endemic areas. Therefore, random sampling was conducted in order to produce precise estimates of prevalence. For finer targeting of schistosomiasis control, each locality was subdivided into one to three different ecological zones based on proximity to water bodies (near, less than 1 km; medium, 1–5 km; far, greater than 5 km), using information about distances provided by government officials of the state Ministry of Health. An ecological zone was defined as areas located within a similar distance from bodies of water within a locality. Some localities had only one or two ecological zones.

We selected five schools from each ecological zone. Probability-proportional-to-size (PPS) sampling was employed for selecting schools. Schools located in insecure areas were excluded. After selecting schools, 60 students were sampled from each school, reflecting an addition of 10 students above the WHO recommendations due to an anticipated 16% non-response rate. We selected 20 students each from grades 2, 4 and 6 using systematic sampling. Global positioning system devices were used to record the coordinates of the selected schools. The survey was conducted from December 2016 to March 2017.

### Data collection

Stool and urine samples were collected from the selected students and processed within 24 h. Eggs of *S. mansoni* and IHs were examined by reading two smears of stool using the Kato-Katz technique. The centrifugation method was used to examine *S. haematobium* eggs in urine samples. A total of 655 people were temporarily employed for the survey, most of whom were government officials or experienced laboratory technicians in state-run hospitals. Participants were interviewed about their behaviours and the water source and type of sanitation used in their household. School-level latrine and water sources were directly observed.

### Detection of schistosomiasis and helminthiasis, quality assurance

For *S. haematobium*, the eggs were double-counted within an hour following centrifugation by two laboratory technicians. The intensity of the infection was estimated by counting the number of eggs of *S. haematobium* per 10 ml of urine and classified as either a light or heavy infection. For *S. mansoni*, the slides were observed under a microscope by two technicians. Each team was given a training module (WHO Benchmark) that included images of the various parasite eggs expected. To ensure high quality of the laboratory work, internal and external quality assurance mechanisms were employed. Central-level supervisors comprising senior laboratory technicians and government officials were deployed and re-examined 10% of slides on a daily basis for internal quality assurance. A parasitologist and molecular biologist (Professor, PhD) from Al Neelain University and two senior technologists (PhD) from the Blue Nile Institute, University of Gezira, were contracted and re-checked 5% of slides for validation as external quality assurance.

### Data analysis

We used tablet PCs (SM-Galaxy T350NZAAXAR, Samsung, Seoul, Korea; MediaPad T1 7.0, Huawei, Shenzhen, China) to enter the laboratory and interview results. The main purpose of using tablet PCs was to help central supervisors conduct real-time monitoring of the survey on a daily basis. State coordinators submitted all the data, which were subsequently exported into SPSS. National coordinators monitored ongoing progress and analysed the preliminary results on a daily basis. Geographical coordinates were collected by either the PCs when connected to the internet or a handheld GPS device (eTrex, Garmin International, Olathe, KS, USA). We used STATA v.13 (StataCorp Llc, College Station, TX, USA) for statistical analyses in this study. Sample weighting was applied by state according to the sex ratio and population size of each district. We calculated the number of infected people in each state by assigning weights for the sex ratio and population proportion of each district within the state, assuming that the prevalence in schoolchildren represents the prevalence in all ages of the population. It was possible to estimate the infected population because we randomly sampled the schools and students, and the sample size was large enough to estimate the precise prevalence in each state [23]. Geometric mean intensities of infection with 95% CIs for the various parasite species were calculated, including all children examined. We categorized individual infections as heavy (≥ 50 eggs/10 ml of urine) and light (< 50 eggs/10 ml of urine) infections for *S. haematobium*, and heavy (≥ 400 epg), moderate (100–399 epg) and light (< 100 epg) infections for *S. mansoni* [25].

Multi-level mixed-effect models were used to investigate the relationship between the prevalence of infections and risk factors, including improved water or latrine status at the household or school level. Geographical information system software (QGIS v.3.2; QGIS Development Team) was used to plot the prevalence of the infections and sanitation coverage for states or districts on a map. In addition, the MDA target population and its cost were calculated both at the district and ecological zone levels, and we explored the potential coverage of MDA depending on its administrative unit (i.e. district or ecological zone level). When calculating the target population of the MDA intervention, 3% and 5% were used as thresholds for school-aged children (SAC), and 8% and 15% for community-wide interventions. The values of 3% and 8% were actually used for MDA, targeting SAC and community-wide treatment, respectively, in Sudan in 2017–2018, and 5% and 15% were recommended by Nathan et al. [26, 27] as optimal levels for cost-effectiveness. The cost-effectiveness of a one-time MDA intervention with 75% coverage was estimated at the district and ecological zone levels using parameter values presented in previous studies [26, 27]. Disability was calculated on the basis of updated disability weights, and an equal disability weight was applied to all infection intensities [28].

## Results

### General characteristics of participants

A total of 1772 schools were surveyed from 390 ecological zones in 183 localities. A total of 105,167 students were interviewed, including more boys (55%) than girls (45%) (Table 1). Urine and stool samples were collected from 100,726 and 96,634 students, respectively. The difference between the number of interviewees and of the samples was caused by the fact that some students who were interviewed did not submit urine or stool samples because they could not urinate or defecate on the same date of the survey.

**Table 1:** General characteristics of participants

State	Number of localities (districts)	No. of ecological zones	No. of schools	No. of students^a^					Mean age of students ± SD	
				Boys		Girls		Total	Boys	Girls
				*n*	%	*n*	%			
Red Sea	10	15	55	1922	57.5	1418	42.5	3340	10.01 ± 2.27	10.08 ± 2.08
River Nile	7	16	58	2076	56.2	1619	43.8	3695	10.42 ± 2.06	10.12 ± 2.02
Kassala	11	12	60	2184	60.8	1411	39.2	3595	11.02 ± 2.54	10.43 ± 2.09
Khartoum	7	20	95	3656	60.5	2385	39.5	6041	11.33 ± 2.97	9.88 ± 1.94
Al Jazirah	8	22	106	3391	53.8	2916	46.2	6307	10.59 ± 2.26	10.19 ± 2.06
Al Qadarif	12	21	103	2564	49.3	2640	50.7	5204	11.33 ± 2.43	10.79 ± 2.22
Sennar	7	14	51	1687	55.0	1378	45.0	3065	10.84 ± 2.10	10.53 ± 2.00
Blue Nile	7	15	68	2222	57.3	1657	42.7	3879	11.07 ± 2.37	11.01 ± 2.29
White Nile	9	25	121	3757	49.7	3805	50.3	7562	10.76 ± 2.13	10.43 ± 1.95
West Kordofan	14	38	183	6254	58.9	4371	41.1	10,625	11.31 ± 2.43	10.88 ± 2.27
North Kordofan	8	13	62	2159	59.3	1481	40.7	3640	11.12 ± 2.28	10.54 ± 2.13
South Kordofan	14	36	161	4846	51.2	4615	48.8	9461	10.92 ± 2.24	10.65 ± 2.12
Northern	7	14	65	1872	48.8	1968	51.2	3840	10.55 ± 2.11	10.08 ± 1.93
Central Darfur	6	21	74	2300	52.2	2109	47.8	4409	11.71 ± 2.69	11.30 ± 2.44
East Darfur	9	16	75	2570	54.6	2135	45.4	4705	11.60 ± 2.42	11.25 ± 2.24
West Darfur	8	15	72	2371	55.2	1928	44.8	4299	11.53 ± 2.26	11.17 ± 2.35
North Darfur	18	24	116	3854	56.7	2941	43.3	6795	11.09 ± 2.42	10.78 ± 2.29
South Darfur	21	53	247	8258	56.2	6447	43.8	14,705	11.42 ± 2.35	11.24 ± 2.28
Total	183	390	1772	57,943	55.1	47,224	44.9	105,167	11.10 ± 2.41	10.70 ± 2.21

^a^The number included all the children interviewed; hence, it is larger than the number of children whose stool or urine samples were examined

### The prevalence of schistosomiasis and infected populations

The prevalence and infected populations are shown together with the weighted population prevalence in Table 2 (see Additional file 1: Tables S1, S2 for prevalence by grade and IH prevalence). The most prevalent helminth was *S. haematobium* and 5272 (5.2%) children were found to be infected in 411 schools. The prevalence ranged from 0 to 57.5% across the 18 states at the school level. The most endemic areas were concentrated in East and South Darfur States, with a prevalence of 25.23 and 13.91%, respectively, for *S. haematobium*.

**Table 2:** Prevalence of *Schistosoma haematobium* and *Schistosoma mansoni* and infected population by state

State	*S. haematobium*					*S. mansoni*				
	*n*/*N*^a^	Geometric mean intensity (95% CI)	Population prevalence (95% CI)	Weighted population prevalence^b^ (95% CI)	Infected population (× 1000)^c^	*n*/*N*^a^	Geometric mean intensity (95% CI)	Population prevalence (95% CI)	Weighted population prevalence^b^ (95% CI)	Infected population (× 1000)^c^
Red Sea	3/2946	17.8 (0.1–3980.6)	0.1 (0.0–0.3)	0.1 (0.0–0.3)	1.5 (0.5–4.9)	0/2425	-	0	0	0
River Nile	50/3258	12.0 (7.9–18.4)	1.5 (1.1–2.0)	1.9 (1.4–2.5)	21.1 (15.7–28.3)	4/2988	102.1 (1.9–5636.4)	0.13 (0.05–0.36)	0.13 (0.05–0.37)	1.5 (0.6–4.1)
Kassala	6/3484	21.7 (3.7–126.5)	0.2 (0.1–0.4)	0.2 (0.1–0.3)	2.7 (1.3–6.1)	202/3441	43.3 (36.4–51.5)	5.87 (5.13–6.71)	4.54 (3.95–5.21)	81.3 (70.7–93.2)
Khartoum	299/5958	14.1 (11.6–17.2)	5.0 (4.5–5.6)	4.3 (3.8–4.8)	319.7 (284.1–359.0)	84/5514	37.0 (27.7–49.3)	1.52 (1.23–1.88)	1.27 (1.00–1.60)	94.2 (74.2–118.7)
Al Jazirah	30/5931	7.8 (4.8–12.6)	0.5 (0.3–0.7)	0.6 (0.4–0.8)	26.7 (18.4–38.2)	151/5710	58.5 (51.4–66.7)	2.64 (2.26–3.09)	2.58 (2.20–3.02)	118.7 (101.2–138.9)
Al Qadarif	82/6163	26.1 (16.0–42.5)	1.3 (1.1–1.6)	1.3 (0.9–1.7)	20.8 (15.6–28.0)	85/5821	2.3 (1.6–3.2)	0.15 (0.12–0.18)	1.56 (1.16–2.09)	25.8 (19.2–34.6)
Sennar	17/2804	10.9 (6.5–18.3)	0.6 (0.4–1.0)	0.7 (0.4–1.2)	9.1 (5.3–15.8)	18/2763	39.6 (24.3–64.4)	0.65 (0.41–1.03)	1.11 (0.67–1.83)	14.3 (8.6–23.5)
Blue Nile	99/3803	18.7 (15.6–22.4)	2.6 (2.1–3.2)	3.4 (2.8–4.2)	36.6 (29.8–45.0)	10/3709	16.4 (6.1–44.0)	0.3 (0.15–0.50)	0.32 (0.16–0.63)	3.4 (1.7–6.7)
White Nile	380/7264	11.0 (9.6–12.5)	5.2 (4.7–5.8)	3.7 (3.2–4.2)	75.0 (65.6–85.8)	5/6579	16.8 (3.7–76.9)	0.08 (0.03–0.18)	0.10 (0.04–0.25)	2.0 (0.8–5.1)
West Kordofan	419/10,459	7.4 (6.6–8.4)	4.0 (3.6–4.4)	3.6 (3.3–4.0)	63.5 (57.6–70.2)	2/10,190	26.9 (23.5–30.7)	0.02 (0.00–0.08)	0.03 (0.01–0.12)	0.5 (0.2–2.1)
North Kordofan	95/3626	6.0 (4.7–7.6)	2.6 (2.1–3.2)	2.3 (1.9–2.9)	47.3 (37.7–59.1)	1/3578	10	0.03 (0.00–0.20)	0.02 (0.00–0.11)	0.4 (0–2.2)
South Kordofan	469/8572	7.4 (6.6–8.3)	5.5 (5.0–6.0)	5.2 (4.7–5.7)	88.6 (80.4–97.4)	3/8402	4.3 (0.0–2311.0)	0.04 (0.01–0.11)	0.03 (0.01–0.09)	0.5 (0.7–4.3)
Northern	17/3762	31.9 (19.2–52.8)	0.5 (0.3–0.7)	0.2 (0.1–0.4)	1.7 (1.0–2.9)	1/3578	9	0.03 (0.00–0.20)	0.02 (0.00–0.15)	0.1 (0–1.1)
Central Darfur	152/3318	3.9 (3.3–4.5)	4.6 (3.9–5.3)	5.6 (4.7–6.6)	58.9 (49.8–69.7)	1/3175	24	0.03 (0.00–0.22)	0.01 (0.00–0.09)	0.1 (0–0.9)
East Darfur	797/4348	30.3 (27.8–33.1)	18.3 (17.2–19.5)	25.2 (23.7–26.9)	251.2 (235.6–267.5)	14/4252	18.5 (9.0–37.9)	0.33 (0.20–0.56)	0.40 (0.22–0.72)	4.0 (2.2–7.2)
West Darfur	62/4063	17.4 (11.5–26.4)	1.5 (1.2–2.0)	1.7 (1.3–2.2)	17.6 (13.4–23.1)	12/3944	74.4 (17.7–313.1)	0.30 (0.17–0.54)	0.21 (0.11–0.39)	2.2 (1.1–4.1)
North Darfur	80/6601	4.4 (3.6–5.5)	1.2 (1.0–1.5)	0.7 (0.5–0.9)	1.7 (1.3–2.3)	17/6562	3.1 (1.6–6.1)	0.26 (0.16–0.42)	0.23 (0.13–0.38)	6.1 (3.5–10.1)
South Darfur	2215/14,366	55.8 (51.7–60.2)	15.4 (14.8–16.0)	13.9 (13.3–14.6)	494.5 (472.1–518.0)	3/14,048	16.2 (0.8–333.4)	0.02 (0.01–0.06)	0.01 (0.00–0.05)	0.4 (0–1.8)
Total	5272/100,726	23.5 (22.4–24.7)	5.2 (5.1–5.4)	4.0 (3.6–4.5)	1538.2 (1385.0–1721.4)	613/96,679	26.9 (23.5–30.7)	0.06 (0.05–0.07)	0.06 (0.05–0.07)	355.5 (284.6–458.7)

^a^*n*, number of children infected; *N*, number of children examined (the values of *N* in Table 2 are slightly different from those in Table 3 because parasite counts were missing for some infected children

^b^Populations of districts in each state were weighted

^c^Population unit: 1000 people

**Fig. 1:**
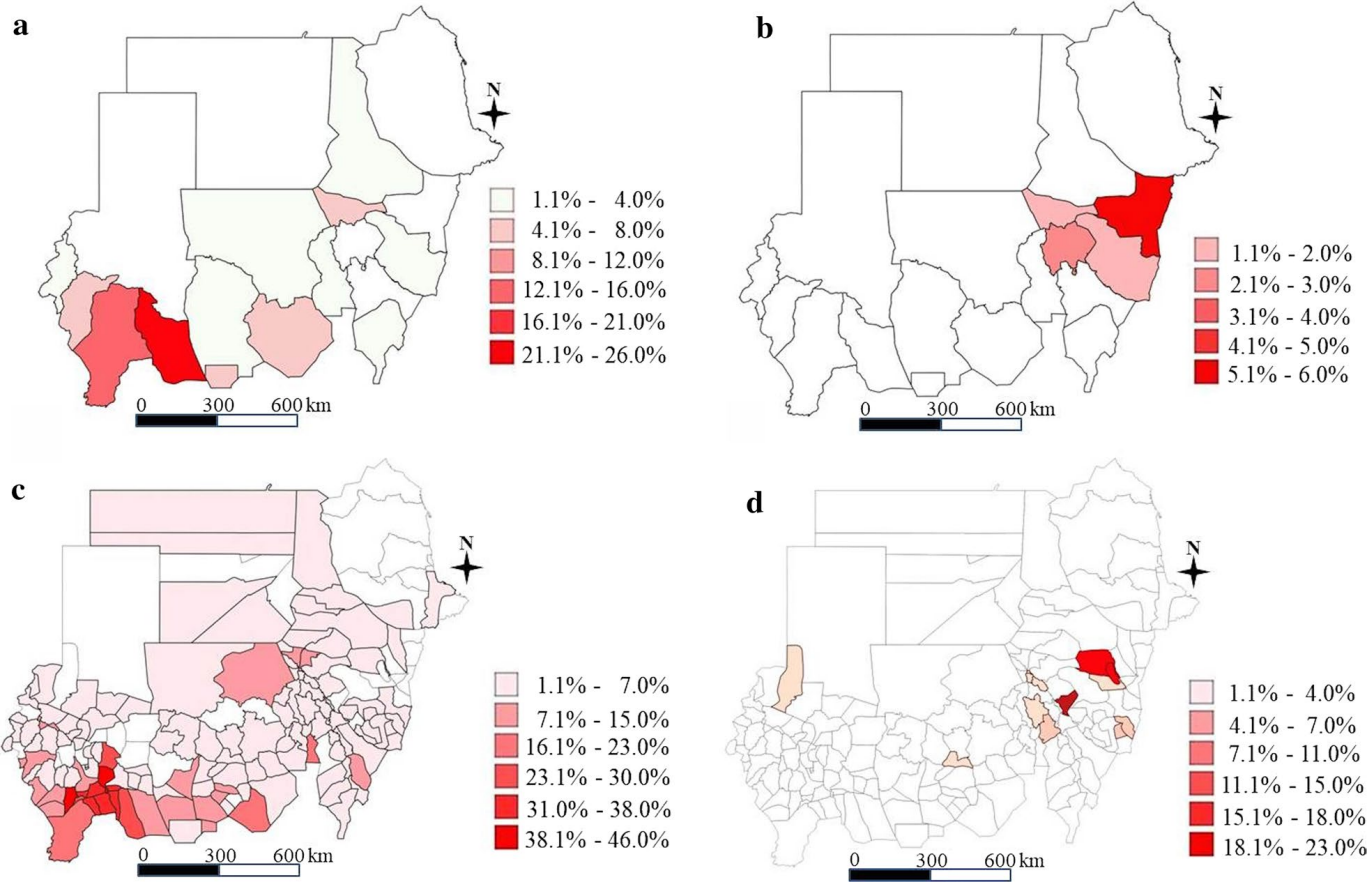
Schistosomiasis prevalence. **a** *Schistosoma haematobium* at state level. **b** *Schistosoma mansoni* at state level. **c** *Schistosoma haematobium* at district level. **d** *Schistosoma mansoni* at district level

The geographical distribution of schistosomiasis is depicted in Fig. 1 at the district and state level (Additional file 2: Figure S1 for the prevalence of IHs). The prevalence, arranged by intensity in the 18 states, is shown in Table 3, and the range of prevalence at the district and ecological zone level is presented in Additional file 1: Tables S3, S4. The egg counts ranged from 6.51 to 173.74 eggs/10 ml of urine for *S. haematobium*. The egg counts of *S. mansoni* ranged from 6.35 to 326.92 eggs per gram of stool across states. Kassala State was the most heavily infected with *S. mansoni*. IHs were detected in 5286 children in all 390 ecological zones of the 18 states, mostly comprising *Hymenolepis nana*, and the STH prevalence was very low (0–0.3%).

**Table 3:** Prevalence of *Schistosoma haematobium* and *Schistosoma mansoni* by intensity

State	*S. haematobium*				*S. mansoni*					
	Light infection (< 50 eggs/10 ml)		Heavy infection (≥ 50 eggs/10 ml)		Light infection (< 100 epg)		Moderate infection (100–399 epg)		Heavy infection (≥ 400 epg)	
	*n*/*N*^a^	%	*n*/*N*^a^	%	*n*/*N*^a^	%	*n*/*N*^a^	%	*n*/*N*^a^	%
Red Sea	2/2944	0.07	1/2944	0.03	0/2425	0.00	0/2425	0.00	0/2425	0.00
River Nile	42/3216	1.31	8/3216	0.25	2/2986	0.07	4/2986	0.13	2/2986	0.07
Kassala	4/3480	0.11	2/3480	0.06	161/3244	4.96	202/3244	6.23	5/3244	0.15
Khartoum	228/5730	3.98	71/5730	0.12	72/5434	1.32	84/5434	1.55	4/5434	0.07
Al Jazirah	27/5904	0.46	3/5904	0.05	126/5563	2.26	151/5563	2.71	4/5563	0.07
Al Qadarif	49/6114	0.80	33/6114	0.54	82/5736	1.43	85/5736	1.48	0/5736	0.00
Sennar	17/2787	0.61	0/2787	0.00	16/2745	0.58	18/2745	0.66	0/2745	0.00
Blue Nile	88/3715	2.37	11/3715	0.30	9/3699	0.24	10/3699	0.27	0/3699	0.00
White Nile	331/6933	4.77	49/6933	0.71	5/6574	0.08	5/6574	0.08	0/6574	0.00
West Kordofan	386/10,073	3.83	33/10,073	0.33	2/10,188	0.02	2/10,188	0.02	0/10,188	0.00
North Kordofan	94/3532	2.66	1/3532	0.03	1/3577	0.03	0/3577	0.00	0/3577	0.00
South Kordofan	435/8173	5.35	34/8137	0.42	3/8399	0.04	3/8399	0.04	0/8399	0.00
Northern	11/3751	0.29	6/3751	0.16	1/3532	0.03	0/3532	0.00	0/3532	0.00
Central Darfur	152/3166	4.80	0/3166	0.00	1/3174	0.03	0/3174	0.00	0/3174	0.00
East Darfur	485/3863	12.56	312/3863	8.08	13/4238	0.31	14/4238	0.33	0/4238	0.00
West Darfur	46/4017	1.15	16/4017	0.40	6/3936	0.15	12/3936	0.30	4/3936	0.10
North Darfur	77/6524	1.18	3/6524	0.05	17/6545	0.26	17/6545	0.26	0/6545	0.00
South Darfur	933/13,084	7.13	1282/13,084	9.79	3/14,045	0.02	3/14,045	0.02	0/14,045	0.00
Total	3407/96,970	3.51	1865/96,970	1.92	520/96,040	0.54	613/96,040	0.64	19/96,040	0.02

^a^*n*, number of children infected; *N*, number of children examined (the *N* in this table is different from that in Table 2 because the parasite count was missing for some infected children

### Associations between schistosomiasis prevalence and risk factors

**Fig. 2:**
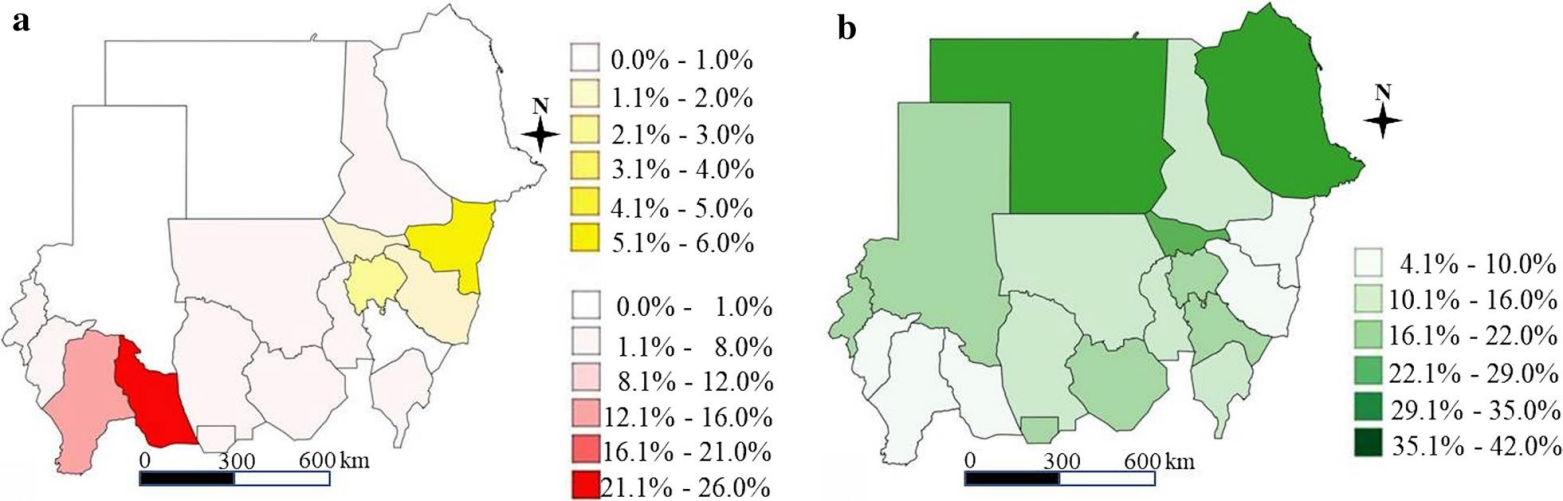
Schistosomiasis prevalence and improved latrine coverage. **a** Schistosomiasis prevalence (red: *S. haematobium* prevalence; yellow: *S. mansoni* prevalence). **b** Improved latrine coverage (green: improved latrine coverage). An improved latrine was defined as a plush toilet or ventilated improved pit latrine

**Fig. 3:**
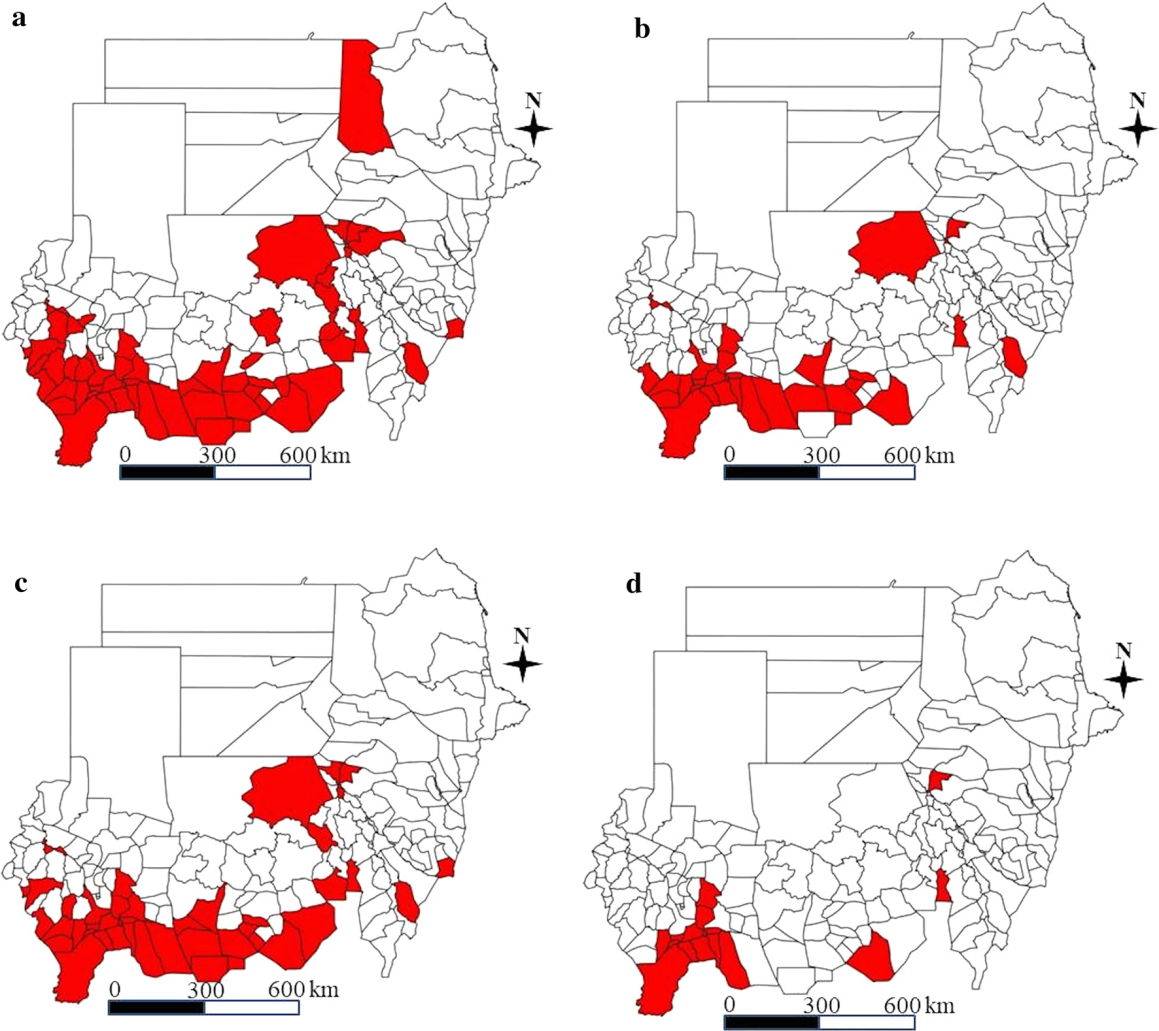
MDA target areas by a different threshold. **a** MDA target areas for school-aged children at 3% prevalence threshold. **b** MDA target areas for all community people at 8% prevalence threshold. **c** MDA target areas for school-aged children at 5% prevalence threshold. **d** MDA target areas for all community people at 15% prevalence threshold (red color: MDA target area)

Table 4 shows the associations between schistosomiasis prevalence and risk factors such as the presence of an unprotected water source or unimproved latrine (simple pit) at both the household and school levels, and open defecation and contact with water at the individual level. Of particular note is that participants with a latrine at home or school had lower odds of being infected with schistosomiasis than those without a latrine, and this association was stronger in those with improved latrines (i.e. ventilated improved pit latrines or flush toilets). For example, children living in a house with an improved latrine were less likely to be infected with schistosomiasis (adjusted odds ratio, aOR: 0.45, 95% confidence interval, CI: 0.41–0.51). Similarly, children attending schools with an improved latrine had lower odds of schistosomiasis infection (aOR: 0.75, 95% CI: 0.70–0.81). Open defecation (aOR: 1.50, 95% CI: 1.35–1.66) and routinely coming into contact with water bodies such as rivers, streams and irrigation canals (aOR: 2.96, 95% CI: 2.79–3.15) were highly associated with schistosomiasis prevalence. Sex was found to be another significant risk factor (aOR for females: 0.63, 95% CI: 0.58–0.69). Figure 2 shows a striking contrast between schistosomiasis prevalence and improved latrine coverage. Figure 3 demonstrates the MDA target coverage depending on the prevalence threshold and Table 5 shows the size of the population that would benefit from MDA interventions by each MDA unit (Additional file 1: Tables S5–S8 for the MDA target areas).

**Table 4:** Association between risk factors and schistosomiasis prevalence (*S. haematobium* or *S. mansoni*)

Risk factors		% (*n*/*N*)	Adjusted OR^a^ (95% CI)	*P*-value
Latrine (household level)	Improved	2.9 (470/16,111)	0.45 (0.41–0.51)	< 0.001
	Unimproved	6.1 (3805/62,507)	0.88 (0.82–0.93)	< 0.001
	No latrine	7.2 (1495/20,779)	ref	
Latrine (school level)	Improved	4.9 (2307/46,813)	0.75 (0.70–0.81)	< 0.001
	Unimproved	5.8 (1701/29,180)	0.82 (0.77–0.89)	< 0.001
	No latrine	7.3 (1825/25,090)	ref	
Open defecation	Yes	7.4 (1394/18,796)	1.50 (1.35–1.66)	< 0.001
	No	5.4 (4439/82,287)	ref	
Water (household level)	Improved	5.7 (5139/90,444)	0.92 (0.84–1.02)	0.13
	Unimproved	6.5 (694/10,639)	ref	
Water (school level)	Improved	5.6 (4727/84,912)	1.05 (0.96–1.14)	0.29
	Unimproved	6.8 (1106/16,171)	ref	
Sex	Female	4.3 (1944/45,280)	0.63 (0.58–0.69)	< 0.001
	Male	7.1 (3826/54,117)	ref	
Routine water contact (contact with water bodies more than 2 times a week^b^)	Yes	10.1 (3866/38,445)	2.96 (2.79–3.15)	< 0.001
	No	3.1 (1904/60,952)	ref	
Water contact type	Fetching water	9.9 (1355/13,718)	1.35 (1.26–1.44)	< 0.001
	Bathing	13.3 (1700/12,770)	1.70 (1.59–1.81)	< 0.001
	Washing clothes	14.3 (1013/7060)	1.94 (1.80–2.10)	< 0.001
	Farming	10.2 (241/2362)	1.11 (0.96–1.28)	< 0.001
	Swimming	11.9 (1909/16,034)	1.82 (1.71–1.94)	< 0.001
	Watering livestock	20.0 (1068/5348)	2.32 (2.14–2.51)	< 0.001
	Fishing	11.2 (62/552)	0.89 (0.67–1.18)	0.42
	No routine water contact	3.1 (1904/60,952)	ref	

^a^Latrine (household/school): adjusted for age, sex, water (household, school), latrine (school/household); water (household/school): adjusted for age, sex, latrine (household, school), water (school/household); open defecation: adjusted for age, sex, water (household, school), latrine (school); sex: adjusted for water (household, school), latrine (household, school); water contact: adjusted for age, sex, water (household, school), latrine (household, school)

^b^River, stream, lake, irrigation canal, reservoir

**Table 5:** Differences in population coverage by MDA intervention between locality and ecological zone levels

MDA type	Threshold (%)	MDA unit		No. of target areas	100% coverage			75% coverage		
					Population	MDA cost (US$)		Population	MDA cost (US$)	
						1 year	5 years		1 year	5 years
Community-wide mass treatment	8.0	District		33	5,597,983	9,740,490	48,702,452	4,198,487	7,305,368	36,526,839
		EZ	Additional	17	2,206,414	3,839,160	19,195,800	1,654,810	2,879,370	14,396,850
			Unnecessary	17	− 1,657,006	− 2,883,190	− 14,415,949	− 1,242,754	− 2,162,392	− 10,811,962
			Total		6,147,391	10,696,461	53,482,303	4,610,543	8,022,345	40,111,727
		Difference			549,408	955,970	4,779,851	412,056	716,978	3,584,888
	15	District		14	2,008,024	3,493,962	17,469,809	1,506,018	2,620,471	13,102,357
		EZ	Additional	12	943,397	1,641,510	8,207,550	707,547	1,231,132	6,155,662
			Unnecessary	2	− 282,602	− 491,727	− 2,458,637	− 211,952	− 368,796	− 1,843,978
			Total		2,668,819	4,643,744	23,218,721	2,001,614	3,482,808	17,414,041
		Difference			660,795	1,149,782	5,748,912	495,596	862,337	4,311,684
Mass treatment for school-aged children	3	District		56	3,733,702	6,496,641	32,483,207	2,800,276	4,872,481	24,362,405
		EZ	Additional	8	202,500	352,349	1,761,747	151,875	264,262	1,321,310
			Unnecessary	32	− 900,103	− 1,566,179	− 7,830,895	− 675,077	− 1,174,634	− 5,873,171
			Total		3,036,099	5,282,812	26,414,058	2,277,074	3,962,109	19,810,544
		Difference			− 697,603	− 1,213,830	− 6,069,148	− 523,202	− 910,372	− 4,551,861
	5	District		41	2,625,068	4,567,618	22,838,092	1,968,801	3,425,714	17,128,569
		EZ	Additional	10	322,228	560,677	2,803,384	241,671	420,508	2,102,538
			Unnecessary	20	− 380,187	− 661,525	− 3,307,625	− 285,140	− 496,144	− 2,480,718
			Total		2,567,109	4,466,770	22,333,851	1,925,332	3,350,078	16,750,389
		Difference			− 57,959	− 100,848	− 504,241	− 43,469	− 75,636	− 378,181

*Abbreviation*: EZ, ecological zone

### Population coverage of MDA

**Fig. 4:**
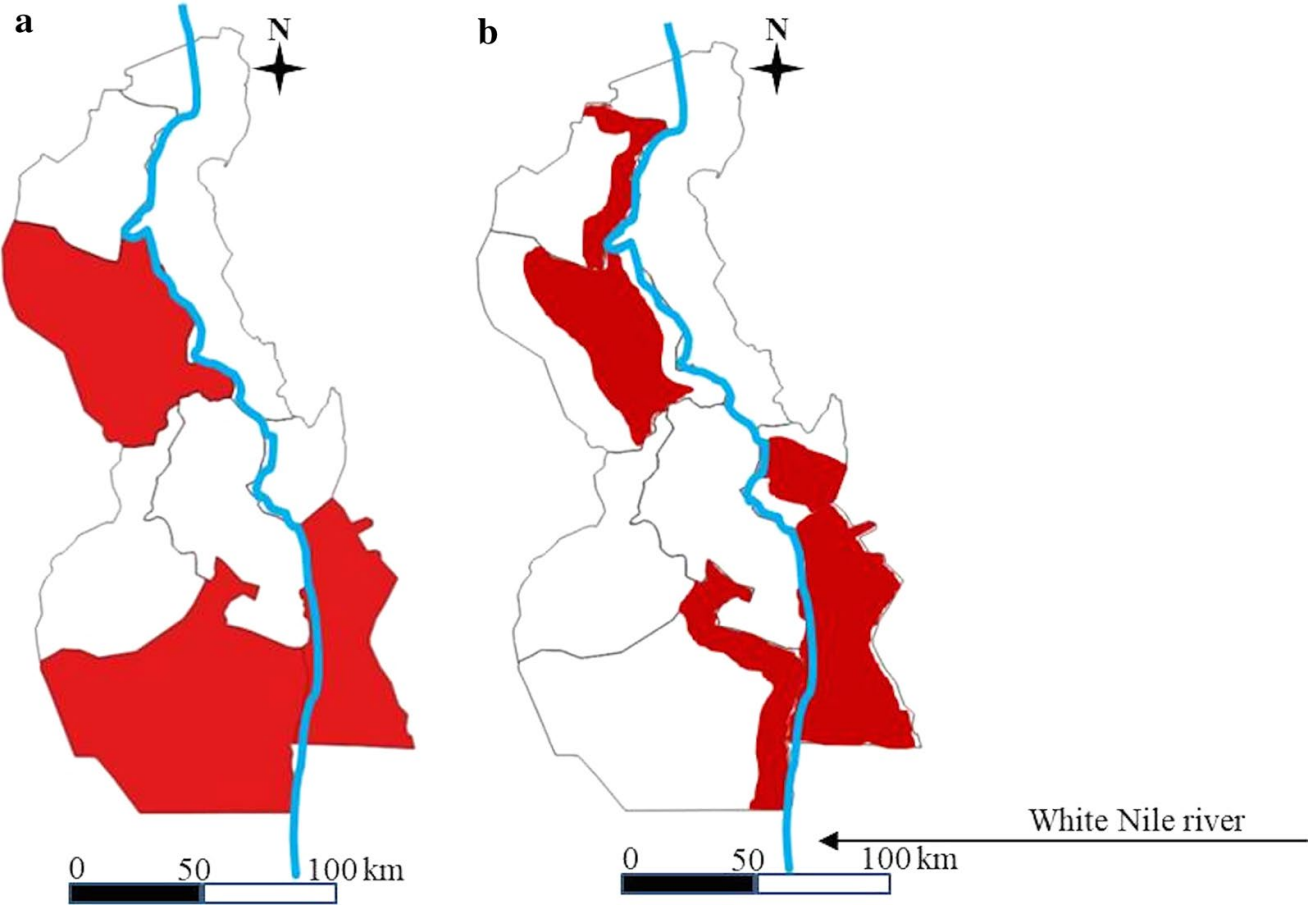
A comparison of MDA target areas by implementation unit in White Nile State. **a** District level. **b** Ecological zone (sub-district) level (red color: MDA target area)

In community-wide mass treatment at the district level with an 8% threshold for schistosomiasis, 2.2 million people would not benefit from MDA interventions with 75% coverage despite high endemicity in their ecological zones, whilst 1.7 million people would receive the MDA intervention unnecessarily. Similarly, if mass treatment targeting school-aged children (SAC) with a 3% threshold was undertaken at the ecological zone level, 0.2 million people who would otherwise be excluded would newly benefit from treatment, and 0.9 million people in whom MDA would be unnecessary would be able to opt out of MDA. Figure 4 demonstrates that the MDA target areas varied considerably depending on the implementation unit.

### Cost-effectiveness of a one-time MDA intervention

Table 6 shows the cost-effectiveness of MDA interventions at the ecological zone and district levels, suggesting that ecological zone-level interventions are more cost-effective than district-level interventions under all circumstances, both for community-wide and SAC-only MDA programmes.

**Table 6:** Cost-effectiveness of a one-time MDA intervention at the district and ecological zone levels (75% coverage)

MDA strategy	Level of MDA	Cost^a^ (US$)	DALY^b^ (averted)	ICER (US$ per DALY averted)
Community-wide at 15%	Ecological zone	3,482,808	9057	385
	District	2,620,471	6784	386
Community-wide at 8%	Ecological zone	8,022,345	13,684	586
	District	7,305,368	10,323	708
SAC only (school-aged children) at 5%	Ecological zone	3,350,078	4338	772
	District	3,425,714	3755	912
SAC only (school-aged children) at 3%	Ecological zone	3,962,109	4506	879
	District	4,872,481	3950	1234

^a^Cost of community-based treatment and delivery, US$1.74; cost of school-based treatment and delivery, US$0.74

^b^Disability was calculated on the basis of updated disability weights. An equal disability weight was applied to all infection intensities

*Abbreviations*: DALY, disability-adjusted life-years; ICER, incremental cost-effectiveness ratio

## Discussion

This nationwide survey, conducted more than 30 years after the first national survey by the WHO, determined the geographical distribution of schistosomiasis and other IHs in Sudan, and also revealed important epidemiological findings, particularly related to latrine status and risk behaviours such as open defecation. The overall prevalence was 5.2% for *S. haematobium*, 0.06% for *S. mansoni* and 5.47% for IHs in Sudan. STH prevalence was 0.2%, indicating that it is not important from a public health perspective. These results are consistent with those of recent studies [29, 30] conducted in some states of South Sudan bordering Sudan, where the overall prevalence of *S. haematobium* was 3.0%, and that of *S. mansoni* was 0.2%. Boys tended to be more frequently and heavily infected than girls, which was consistent with prior studies.

This nationwide survey further refined the methodology of existing surveys in Sudan. The geographical foci of schistosomiasis were identified by dividing districts into ecological zones, and sufficient sample sizes and random sampling made it possible to estimate precise prevalence at the state level, which is critical for better design and monitoring of control programmes and their progress.

The results of federal-level and independent quality control demonstrated that the laboratory examinations in this survey were of high quality. Fewer than ten examination results (0.01%) from laboratory technicians were found to be mismatched by federal or independent supervisors. Recruiting experienced experts for state laboratories, most of whom were senior laboratory technicians at state hospitals, led to the high-quality examination results.

It is worth noting that the hyper-endemicity of schistosomiasis was concentrated in fragile and border states. East Darfur (18.3%) and South Darfur (15.4%) States, which border Chad, the Central African Republic and Congo, are the most insecure areas in Sudan due to frequent conflicts, and diverse groups of development partners are working to control and stabilise these areas. Urgent measures should be taken to control the high prevalence of schistosomiasis and to disrupt its transmission in these unstable states. Darfur, South Kordofan, and White Nile States border South Sudan, and migration or human mobilization frequently occurs across these borders [29, 30]. Collaboration between both governments and among development partners working in each country is required for designing and implementing control programmes, and joint efforts are required to disrupt disease transmission and to sustain control effects.

Although this was a cross-sectional study on schistosomiasis and IHs, the results demonstrate the potential contribution of latrine improvement, open defecation eradication and reductions in the likelihood of coming into contact with polluted water. The importance of water and sanitation improvements is well recognised as a key element of NTD control strategies [31]. However, control programmes are often limited to preventive chemotherapy in practice, partly because evidence is lacking regarding the possible impacts of improved water and sanitation on NTD infections [15–17]. The findings of higher odds of infection among children without a household or school latrine and among those who frequently come into contact with contaminated water suggest that mere anthelmintic drug administration neither sufficiently controls or eliminates these diseases nor has a sustained effect. Incorporating WASH components into control programmes will accelerate breaking the transmission cycle and promote more sustained outcomes. This study provides insight into possible ways to design programmes to effectively reduce infections by illustrating the potential of diverse, innovative ways to disrupt the transmission cycle. For instance, building small stations with locally available materials at low cost for doing laundry or watering livestock near rivers or streams could help children to avoid contact with polluted water. In many villages, we observed residents living on both sides of a stream or river crossing it on foot or wading in the water to travel in traditional boats frequently, for purposes including agricultural activities and going to school. Therefore, inter-sectoral collaboration between the WASH and NTD sectors will be vital for designing and implementing control and elimination programmes suitable for each local context [16].

This study also suggests that a tremendous number of people would receive appropriate MDA interventions if those interventions are designed at smaller units than the district level, and the potential to avoid unnecessary mass treatment is no less important. Using the WHO protocol [32], tens of thousands of people living in highly endemic areas would not receive treatment, and other people not in need of treatment would be treated, as was the case in Senegal [18]. The sample size for schistosomiasis was larger than would have been obtained using the WHO methodology; however, this novel method would result in more targeted and cost-effective MDA for schistosomiasis.

We encountered difficulties in accessing target schools in several unstable states, such as Central Darfur, South Kordofan and West Kordofan, where surveys were delayed and some schools had to be replaced with others due to security issues. The parents of five girls’ schools in Red Sea State refused to participate in the survey, due to their opinion that it was unacceptable to submit samples from their daughters, and we thus replaced those schools with others. However, the overall proportion of schools replaced was less than 0.5% of the total number of target schools. This experience underscores the importance of community engagement prior to the survey as a way to increase the likelihood of parents allowing specimens from their children to be collected and examined. Conducting health education campaigns can be a good example of this. This was particularly important in some rural areas of Sudan, where there were rumours that the nationwide survey was intended to harm community members or that the treatment would cause women to be infertile. Extensive efforts were made to recruit a sufficient number of data collectors and laboratory workers, in particular to avoid placing a burden on the existing health system, since most of them were employees in the public sector, either at state hospitals or the state Ministries of Health. By temporarily recruiting 655 experienced people, we completed this large-scale survey within ten weeks. Despite the short period allowed for the survey, the high profile of supervisors for intensive monitoring and supervision made it possible to undertake an intense implementation of nationwide data collection and examination of specimens.

In this study, the use of random sampling may have resulted in slightly lower prevalence estimates. However, a previous study [24] indicated that health workers were not always well-informed about where schistosomiasis was most prevalent, and treatment decisions based on purposively-selected villages therefore did not systematically result in more treatments than those based on randomly-selected villages. Another limitation of this study is that we could not directly observe water sources and latrines at the household level due to time and workforce constraints. However, doing so should be considered for the next round of the survey.

## Conclusions

Our findings provide updated prevalence figures to guide preventive chemotherapy programmes for schistosomiasis and intestinal helminthiasis in Sudan. Schistosomiasis was found to be common among the inhabitants of fragile and conflict-affected areas. We found that MDA interventions would be more cost-effective at the sub-district level than at the district level, and there was a strong association between schistosomiasis prevalence and latrine status, at both the household and school levels. Resource constraints have impeded schistosomiasis and soil-transmitted helminthiasis control and elimination. Estimating the prevalence of these conditions on a solid epidemiological basis will help the Sudanese government and its neighbouring countries develop adequate control and elimination strategies. This study highlights that development partners inside Darfur need to pay attention to the high prevalence of schistosomiasis, and that neighbouring countries should work together to develop adequate control and elimination strategies against schistosomiasis occurring in border areas. Comprehensive approaches surrounding WASH interventions, as well as preventive chemotherapy, may have tremendous potential for schistosomiasis and helminthiasis elimination. Developing innovative ways to avoid contact with contaminated water, such as laundry stations or troughs made with locally available material, is no less important.

## Supplementary information

**Additional file 1: Table S1.** Prevalence of schistosomiasis by grade. **Table S2.** Other helminthiases prevalence. **Table S3.** Schistosomiasis haematobium prevalence by state, locality and ecological zone. **Table S4.** Schistosomiasis mansoni prevalence by state, locality and ecological zone. **Table S5.** MDA target areas (community-wide at 8% a threshold). **Table S6.** MDA target areas (community-wide at a 15% threshold). **Table S7.** MDA target areas (school-aged children at a 3% threshold). **Table S8.** MDA target areas (school-aged children at a 5% threshold).

**Additional file 2: Figure S1.** The prevalence of other intestinal helminthiasis at state level.

## Data Availability

Data supporting the conclusions of this article are provided within the article and its additional files. Raw data cannot be shared publicly because of Sudanese Government policy. Data are available from the Federal Ministry of Health, Sudan (contact Dr Mousab Siddig Elhag, mousabsiddig@gmail.com) for researchers who meet the criteria for access to confidential data.

## Abbreviations

NTD: neglected tropical disease
EZ: ecological zone
MDA: mass drug administration
WASH: water, sanitation and hygiene
STH: soil-transmitted helminthiasis
PPS: probability-proportional-to-size
SAC: school-aged children
IH: intestinal helminthiasis
DALY: disability-adjusted life-year
ICER: incremental cost-effectiveness ratio
