# Supplementary figures and images for: Epidemiological findings and policy implications from the nationwide schistosomiasis and intestinal helminthiasis survey in Sudan
Source: Parasit Vectors. 2019 Sep 5;12:429. doi: 10.1186/s13071-019-3689-z (PMC6728938; doi:10.1186/s13071-019-3689-z)

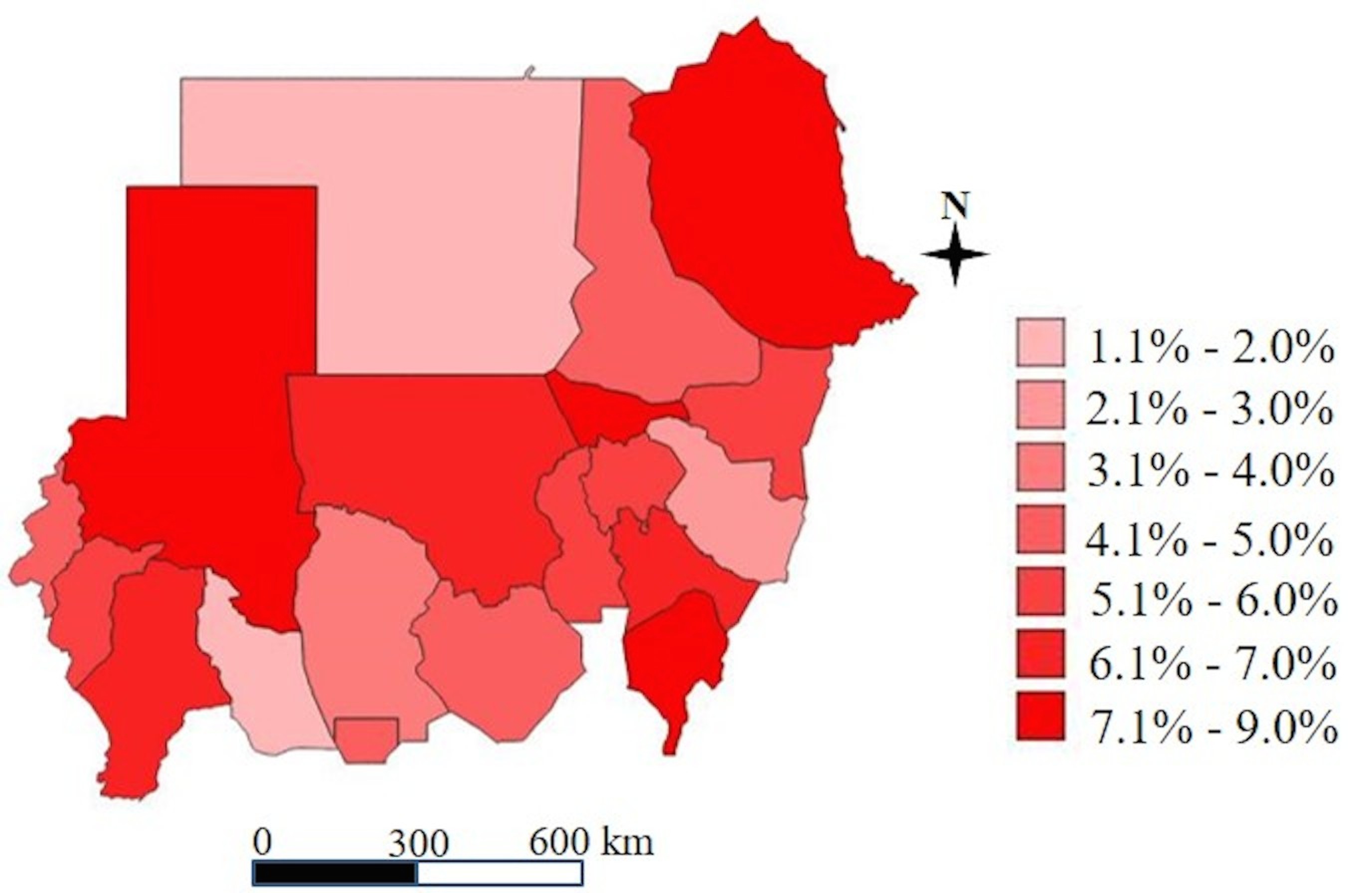

Supplement: Supplementary file 2 — Additional file 2: Figure S1. The prevalence of other intestinal helminthiasis at state level. [file 13071_2019_3689_MOESM2_ESM.jpg]
